# Global and segmental absolute stress myocardial blood flow in prediction of cardiac events: [^15^O] water positron emission tomography study

**DOI:** 10.1007/s00259-020-05093-2

**Published:** 2020-11-11

**Authors:** Esa Harjulahti, Teemu Maaniitty, Wail Nammas, Iida Stenström, Fausto Biancari, Jeroen J. Bax, Juhani Knuuti, Antti Saraste

**Affiliations:** 1 Turku PET Centre, Turku University Hospital and University of Turku, Kiinamyllynkatu 4-8, 20520 Turku, Finland; 2 Heart Center, Turku University Hospital, Turku, Finland; 3 Department of Surgery, University of Turku, Turku, Finland; 4 Department of Surgery, University of Oulu, Oulu, Finland; 5 Department of Cardiology, Leiden University Medical Center, Leiden, The Netherlands

**Keywords:** Positron emission tomography, Myocardial blood flow, Chronic coronary syndromes, Net reclassification improvement

## Abstract

**Purpose:**

We evaluated the value of reduced global and segmental absolute stress myocardial blood flow (sMBF) quantified by [^15^O] water positron emission tomography (PET) for predicting cardiac events in patients with suspected obstructive coronary artery disease (CAD).

**Methods:**

Global and segmental sMBF during adenosine stress were retrospectively quantified in 530 symptomatic patients who underwent [^15^O] water PET for evaluation of coronary stenosis detected by coronary computed tomography angiography.

**Results:**

Cardiovascular death, myocardial infarction, or unstable angina occurred in 28 (5.3%) patients at a 4-year follow-up. Reduced global sMBF was associated with events (area under the receiver operating characteristic curve 0.622, 95% confidence interval (95% CI) 0.538–0.707, *p* = 0.006). Reduced global sMBF (< 2.2 ml/g/min) was found in 22.8%, preserved global sMBF despite segmentally reduced sMBF in 35.3%, and normal sMBF in 41.9% of patients. Compared with normal sMBF, reduced global sMBF was associated with the highest risk of events (adjusted hazard ratio (HR) 6.970, 95% CI 2.271–21.396, *p* = 0.001), whereas segmentally reduced sMBF combined with preserved global MBF predicted an intermediate risk (adjusted HR 3.251, 95% CI 1.030–10.257, *p* = 0.044). The addition of global or segmental reduction of sMBF to clinical risk factors improved risk prediction (net reclassification index 0.498, 95% CI 0.118–0.879, *p* = 0.010, and 0.583, 95% CI 0.203–0.963, *p* = 0.002, respectively).

**Conclusion:**

In symptomatic patients evaluated for suspected obstructive CAD, reduced global sMBF by [^15^O] water PET identifies those at the highest risk of adverse cardiac events, whereas segmental reduction of sMBF with preserved global sMBF is associated with an intermediate event risk.

## Introduction

Clinical practice guidelines recommend non-invasive functional imaging of myocardial ischemia for the detection of obstructive coronary artery disease (CAD) and to inform decisions on revascularization [1]. Myocardial perfusion imaging (MPI) with positron emission tomography (PET) has a high diagnostic accuracy for the identification of obstructive CAD [2–4]. Compared with relative perfusion images, absolute quantification of myocardial blood flow (MBF) by PET may help uncover the extent of CAD in patients with multivessel disease [5–10].

Myocardial ischemia detected by PET is an independent predictor of long-term all-cause mortality and cardiac events [11–13]. Growing evidence shows that globally reduced stress MBF (sMBF) or myocardial flow reserve (MFR) are prognostic markers, providing incremental prognostic value over regional ischemia and other risk predictors [14–20]. However, evidence is currently based mainly on studies using ^82^Rb PET, adding global sMBF or MFR on top of assessment of relative regional perfusion defects rather than comparing the prognostic value of quantified regional and global sMBF.

[^15^O]water PET enables quantification of both regional and global sMBF, and interpretation of [^15^O]water PET studies is solely based on quantitative values. Segmental sMBF ≤ 2.3 ml/g/min accurately detects obstructive CAD defined as FFR < 0.80, both at per-patient and per-vessel levels [10]. Previously, we demonstrated that reduced segmental sMBF quantified by [^15^O]water PET was associated with increased risk of death, myocardial infarction (MI), or unstable angina [21]. Global sMBF integrates the extent and severity of myocardial perfusion abnormalities, but the prognostic value of global sMBF as related to the regionally measured absolute sMBF remains less well established. Moreover, the optimal cutoff value of global sMBF with [^15^O]water PET which best predicts adverse clinical outcomes in patients evaluated for suspected CAD is unknown.

Therefore, we sought to explore the relative value of global and segmental sMBF quantified by [^15^O]water PET for predicting cardiac events in patients with suspected CAD and to identify the cutoff value of global sMBF which best predicts such an outcome.

## Methods

### Study cohort

We retrospectively identified all consecutive patients referred for coronary computed tomography angiography (CTA) in Turku PET Centre due to suspected obstructive CAD from January 2006 to December 2014. The patients had predominantly intermediate pre-test probability of obstructive CAD. It is our routine practice that patients undergo coronary CTA using a hybrid PET-CT scanner and immediately after the coronary CTA, the attending physician makes an initial assessment of the CTA scan to decide whether a PET MPI study is needed. If obstructive CAD is excluded by coronary CTA, no further imaging procedure is performed. In case of the presence of obstructive CAD or if obstructive CAD cannot be excluded by coronary CTA, PET MPI is performed using [^15^O]water during adenosine stress to assess the hemodynamic significance of the stenosis. In the present study, we focused on this group of patients with CTA suggestive of obstructive CAD who subsequently underwent PET MPI. We excluded patients with previously known CAD defined as previous coronary revascularization or obstructive CAD documented as ≥ 50% diameter stenosis by invasive coronary angiography; those referred primarily for reasons other than suspected obstructive CAD, including dilated cardiomyopathy and pre-operative evaluation; and those who did not adhere to the protocol. We also excluded patients with unavailable imaging data. In cases of repeated PET MPI scans during the study period, only the earliest scan was included. The study complies with the Declaration of Helsinki. The Ethics Committee of the Hospital District of Southwest Finland approved the study protocol and waived the need for informed consent by patients for the evaluation of data.

### Coronary CTA and PET image acquisition and interpretation

The coronary CTA and PET MPI procedures have been previously described [21, 22]. Coronary CTA scans were performed using a 64-row hybrid PET-CT scanner (GE Discovery VCT or GE D690, General Electric Medical Systems, Waukesha, WI). Collimation was set at 64 × 0.625 mm, gantry rotation time was 350 ms, tube current 600 to 750 mA, and voltage 100 to 120 kV, depending on the patient size. Scans were performed after an overnight fast. Patients were instructed to abstain from alcohol and caffeine for 24 h before the PET MPI study. In some patients, PET MPI was postponed for days or weeks due to logistic reasons or caffeine use. Before coronary CTA, metoprolol (0 to 30 mg) was given intravenously to achieve a target heart rate of < 60 beats/min. Isosorbide dinitrate aerosol (1.25 mg) was administered. Coronary CTA was performed using intravenously administered low-osmolal iodine contrast agent (60–80 ml; 320–400 mg iodine/ml; injection velocity 4–5 ml/s) followed by a saline flush. Prospectively triggered acquisition was applied whenever feasible. Based on the initial evaluation of coronary CTA findings, a dynamic [^15^O]water PET scan was carried out during adenosine stress using a hybrid PET-CT scanner in the same imaging session, as previously described [21, 22]. Adenosine infusion was started 2 min before the stress PET scan and continued at a rate of 140 μg/kg/min until the scan was complete. [^15^O]water (Radiowater Generator, Hidex Oy, Turku, Finland) was injected as an intravenous bolus (mean injected activity 900–1100 MBq) over 15 s, and dynamic PET acquisition was performed (14 × 5 s, 3 × 10 s, 3 × 20 s, and 4 × 30 s). The values of sMBF were expressed as ml/g/min. The PET data were analyzed quantitatively using the Carimas software (developed at Turku PET Centre, Turku, Finland) [23]. Absolute sMBF was quantified individually for each of the standard 17 myocardial segments according to the recommendations of the American Heart Association, and the global sMBF was calculated as the mean sMBF of the 17 segments [24]. Segments 2 and 3 in the basal septum were excluded from the segmental analysis. The analysis was performed by an experienced physician and recorded in a standardized reporting system. Reduced segmental sMBF was defined as sMBF ≤ 2.3 ml/g/min in ≥ 1 segment based on our previous study [10].

### Data collection and follow-up

Data on cardiovascular risk factors, symptoms, exercise electrocardiography findings, laboratory test results, and medication use were retrospectively collected from electronic medical records. Coronary CTA and PET MPI data were obtained from the institutional imaging database and electronic medical records. Clinical and imaging data were collected blinded to outcomes. The primary clinical outcome was a composite of adverse cardiac events defined as cardiovascular death, MI, or unstable angina. Comprehensive data on the occurrence of all-cause death, MI, and unstable angina until 30 October 2018 were obtained from the registries of the Finnish Institute for Health and Welfare and the Centre for Clinical Informatics of the Turku University Hospital. The clinical events identified from these registries were validated by the investigators using electronic medical records. Data on cardiovascular cause of death were obtained from electronic medical records and confirmed by the investigators. In case of the occurrence of multiple adverse events, cardiovascular death was given priority to MI, and MI was given priority to unstable angina. The Finnish Institute for Health and Welfare gave permission to the retrospective collection of the clinical data.

### Statistical analysis

Continuous variables were reported as the mean and standard deviation (SD) or median [interquartile range]. Missing values were not replaced. Categorical variables were reported as count (percentage). The median follow-up time was 6.2 years (25–75th percentile 4.6–7.6 years). At a 6-year follow-up, 44.5% of the patients were censored by a shorter follow-up. Therefore, we explored the predictors of the primary outcome at 4-year follow-up when only 12.6% of the patients were censored by shorter follow-up.

The chi-square test, Fisher exact test, Mann-Whitney test, and Kaplan-Meier estimates were used for comparison of clinical variables and cardiac events. Receiver operating characteristic (ROC) curve analysis and the Youden’s test were used to identify the cutoff value of global sMBF (increments of 0.1 ml/g/min) which best predicted cardiac events. Cox proportional hazards models were used to identify the predictors of cardiac events at 4 years. The proportional hazard assumption was evaluated using the global test based on Schoenfeld residuals and by inspecting the survival curves. The global test showed that all regression models held the Cox proportional hazards assumption (*p* > 0.10).

In order to explore the prognostic value of global and regional sMBF for cardiac events, we studied 3 approaches: (i) the hazard ratio (HR) of reduced vs. preserved global sMBF, (ii) the HR of any segmental reduction of sMBF (i.e., ≥ 1 segment with sMBF ≤ 2.3 ml/g/min) vs. normal sMBF in all segments, and (iii) the HR of reduced global sMBF or reduced segmental sMBF combined with preserved global sMBF vs. normal sMBF in all segments. To test the incremental prognostic value of reduced global and segmental sMBF over the clinical risk predictors for predicting cardiac events at a 4-year follow-up, the net reclassification improvement methodology of Pencina et al. was applied to calculate the net reclassification improvement index (NRI)—as a continuous estimate—and the integrated discrimination improvement (IDI) for survival data [25]. Statistical significance was set at *p* < 0.05. Statistical analyses were performed using Stata v. 15.1 (StataCorp LLC, TX, USA) and SPSS v. 25.0 (IBM Corporation, NY, USA) statistical software.

## Results

### Patient population

**Fig. 1:**
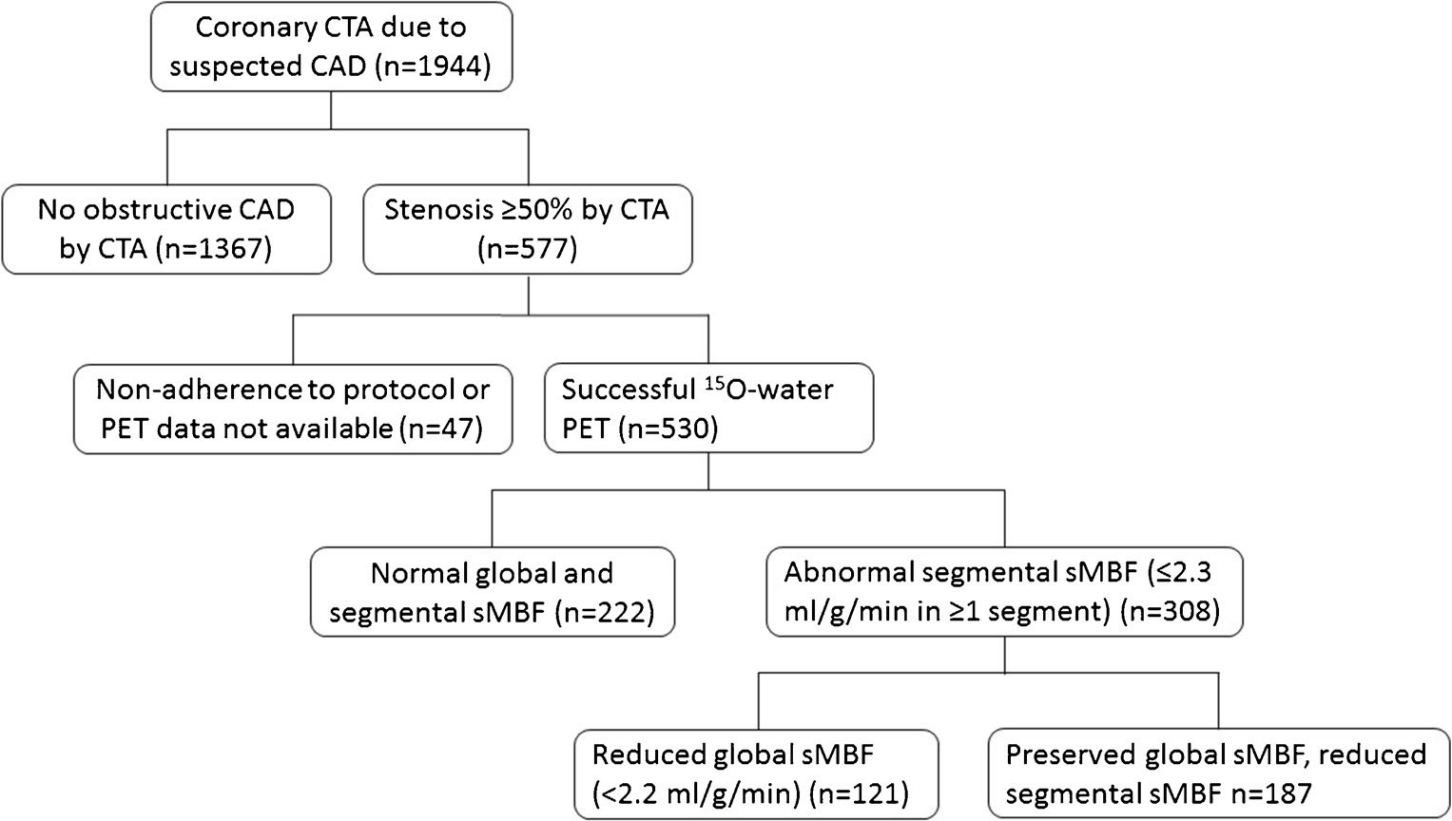
Study flow chart. CAD, coronary artery disease; CTA, computed tomography angiography; PET, positron emission tomography; sMBF, stress myocardial blood flow

We identified 1944 consecutive patients referred for coronary CTA due to suspected obstructive CAD during the study period. We excluded 1367 patients in whom obstructive CAD was ruled out by coronary CTA, and no PET perfusion imaging was performed, as well as 47 patients who did not complete PET testing after finding obstructive CAD on CTA or had PET imaging data not available (Fig. 1). Consequently, the final study cohort consisted of 530 patients who had undergone [^15^O]water PET due to obstructive CAD detected at coronary CTA. The mean age was 65.0 ± 9.2 years, 50.9% were males, and 19.6% had diabetes. The most common presentations were atypical angina (43.0%) and dyspnea (40.9%), whereas 23.8% presented with typical angina. Pretest probability [1] of obstructive CAD before CTA was ≥ 15% in 70.4%, 6–14% in 29.1%, and ≤ 5% in 0.5% of patients. Only one patient had a LV ejection fraction < 40%. Approximately half of the patients were receiving aspirin, statin, beta-blocker, and angiotensin-converting enzyme inhibitor or angiotensin receptor blocker medications at the time of PET scan (Table 1).

**Table 1:** Baseline characteristics

	Total cohort (*n* = 530)	Normal perfusion (*n* = 222)	Preserved global but reduced segmental sMBF (*n* = 187)	Reduced global sMBF (*n* = 121)	*p* value
Characteristic					
Age (years)	65.0 ± 9.2	65.4 ± 9.4	65.1 ± 8.7	64.4 ± 9.6	0.659
Male gender	270 (50.9%)	78 (35.1%)	100 (53.5%)	92 (76.0%)	< 0.001
BMI (kg/m^2^)	28.4 ± 5.2	27.9 ± 4.8	28.5 ± 5.2	29.1 ± 5.8	0.124
Diabetes	104 (19.6%)	35 (15.8%)	46 (24.6%)	23 (19.0%)	0.08
Hypertension	360 (67.9%)	146 (65.8%)	140 (74.9%)	74 (61.2%)	0.02
Hypercholesterolemia	345 (65.1%)	138 (62.2%)	131 (70.1%)	76 (62.8%)	0.208
Current smoker	69 (13%)	26 (11.7%)	22 (11.8%)	21 (17.4%)	0.272
Family history of CAD	224 (42.3%)	103 (46.4%)	72 (38.5%)	49 (40.5%)	0.247
Typical angina	126 (23.8%)	50 (22.5%)	46 (24.6%)	30 (24.8%)	0.847
Atypical angina	228 (43.0%)	96 (43.2%)	82 (43.9%)	50 (41.3%)	0.905
Dyspnea	217 (40.9%)	88 (39.6%)	76 (40.6%)	53 (43.8%)	0.751
Medications					
Lipid-lowering drug	255 (48.1%)	93 (41.9%)	102 (54.5%)	60 (49.6%)	0.03
ACEI/ARB	253 (47.7%)	100 (45.0%)	95 (50.8%)	58 (47.9%)	0.509
Aspirin	281 (53%)	106 (47.7%)	104 (55.6%)	71 (58.7%)	0.103
Beta-blocker	269 (50.8%)	109 (49.1%)	100 (53.5%)	60 (49.6%)	0.649
Calcium antagonist	110 (20.8%)	46 (20.7%)	47 (25.1%)	17 (14.0%)	0.064
Long-acting nitrate	70 (13.2%)	28 (12.6%)	28 (15.0%)	14 (11.6%)	0.650
Diuretic	130 (24.5%)	58 (26.1%)	45 (24.1%)	27 (22.3%)	0.723
Oral anticoagulation	42 (7.9%)	16 (7.2%)	13 (7.0%)	13 (10.7%)	0.424

*ACEI* angiotensin-converting enzyme inhibitor, *ARB* angiotensin receptor blocker, *BMI* body mass index, *CAD* coronary artery disease, *sMBF* stress myocardial blood flow

### Optimal cutoff value of global sMBF for predicting cardiac events

**Fig. 2:**
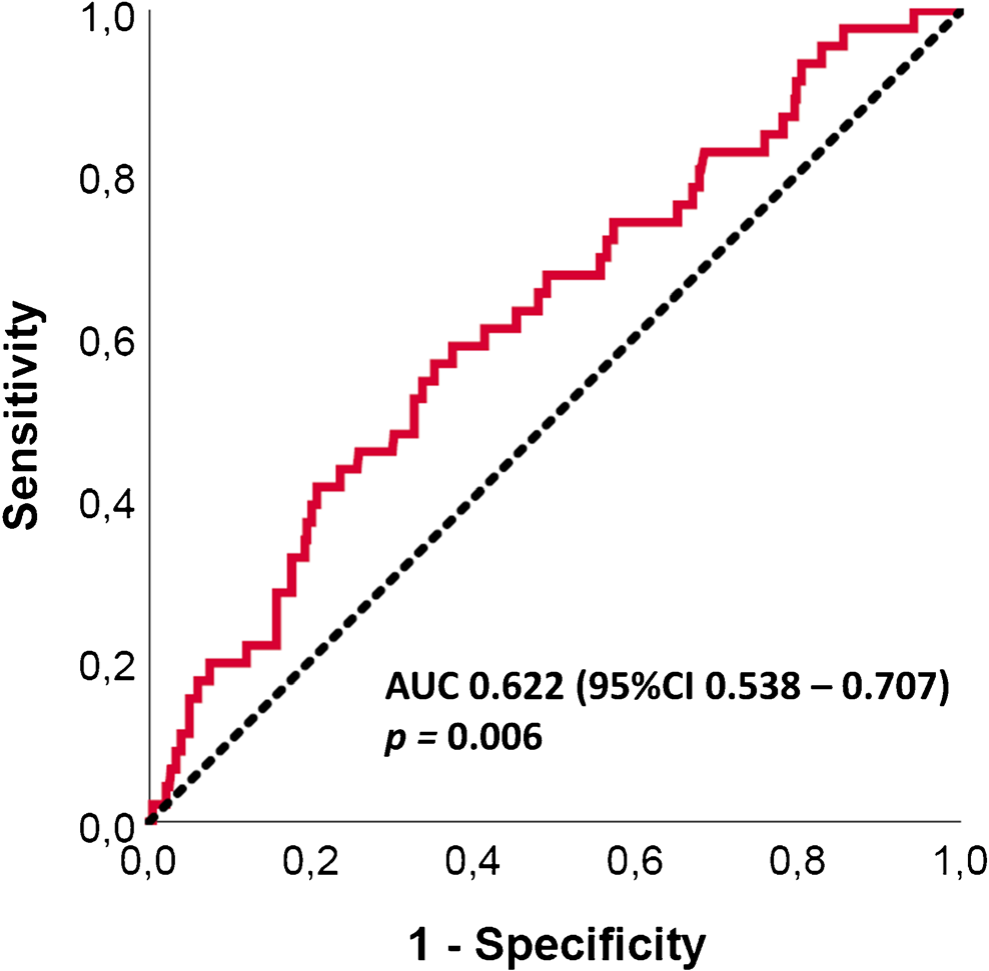
Receiver operating characteristics curve showing the accuracy of global stress myocardial blood flow to predict cardiac events (cardiovascular death, myocardial infarction, or unstable angina pectoris) during a 4-year follow-up

The univariable analysis showed that each 1 unit increase in global sMBF was associated with a HR of 0.551 [95% confidence interval (CI) 0.384–0.790, *p* = 0.001] for cardiac events at 4-year follow-up (Table 2). Based on the ROC curve analysis shown in Fig. 2, the cutoff value of global sMBF, which best identified the high risk of cardiac events, was < 2.2 ml/g/min (AUC 0.622; 95% CI 0.538–0.707, *p* = 0.006, Youden index = 0.198). Using this cutoff value, reduced global sMBF predicted cardiac events with a sensitivity of 41% and a specificity of 79%. In addition to the cutoff value of < 2.2 ml/g/min, a global sMBF of < 2.7 ml/g/min also performed well (Youden index = 0.197) with a higher sensitivity of 57% but a lower specificity of 63%. Moreover, a cutoff value of < 2.3 ml/g/min had a slightly higher sensitivity of 44% and a slightly lower specificity of 76%, but a lower Youden index (0.191).

**Table 2:** Univariable predictors of cardiac events at 4-year follow-up

Variable	HR	95% CI	*p* value
Age (years)	1.072	1.024–1.121	0.003
Male gender	1.312	0.621–2.773	0.477
Diabetes mellitus	2.803	1.313–5.985	0.008
Hypertension	0.616	0.291–1.302	0.204
Dyslipidemia	1.145	0.518–2.532	0.737
Family history of CAD	0.453	0.193–1.067	0.070
Smoking	0.811	0.245–2.687	0.732
Typical angina	1.559	0.706–3.447	0.272
BMI (kg/m^2^)	0.967	0.892–1.048	0.411
eGFR (ml/min/1.73 m^2^)	0.984	0.962–1.006	0.159
Reduced global sMBF	3.191	1.518–6.707	0.002
Reduced segmental sMBF	4.585	1.591–13.216	0.005
Number of segments with reduced sMBF	1.074	1.014–1.138	0.014
Global sMBF (continuous variable, ml/g/min)	0.551	0.384–0.790	0.001

*BMI* body mass index, *CAD* coronary artery disease, *CI* confidence interval, *eGFR* estimated glomerular filtration rate, *HR* hazard ratio, *sMBF* stress myocardial blood flow

Out of 530 patients, 121 (22.8%) patients had reduced global sMBF (< 2.2 ml/g/min), and 409 (77.2%) had preserved global sMBF. Out of these 409 patients with preserved global sMBF, 187 (35.3%) patients had reduced segmental sMBF (≥ 1 segment with ≤ 2.3 ml/g/min), whereas 222 (41.9%) had normal sMBF (> 2.3 ml/g/min) in all segments. Clinical characteristics of the whole cohort as well as patients with normal sMBF in all segments, those with preserved global sMBF despite reduced segmental sMBF, and those with reduced global sMBF are shown in Table 1.

The median [interquartile range] global sMBF for the whole cohort was 3.0 [1.5] ml/g/min. It was 3.9 [1.2] ml/g/min in those who had normal sMBF in all segments. In patients with reduced global sMBF, the median global sMBF was 1.7 [0.8] ml/g/min, whereas it was 2.8 [0.6] ml/g/min in patients with reduced segmental sMBF despite a preserved global sMBF.

### Cardiac events according to global and segmental sMBF

At 4 years, the primary outcome occurred in 28 (5.3%) patients, including 7 cardiovascular deaths, 14 MIs (two of which were followed by cardiovascular death), and 9 unstable angina episodes. In patients with reduced global sMBF, there were 2 deaths, 4 MIs, and 7 unstable angina episodes. In patients with preserved global sMBF despite reduced segmental sMBF, there were 2 deaths, 7 MIs, and 2 unstable angina episodes. In patients with normal sMBF, there were 2 deaths and 3 MIs. When obstructive CAD was ruled out by coronary CTA alone and PET MPI was not performed, the primary outcome occurred in 1.6% (*p* < 0.001 vs. patients with MPI) of patients at 4 years.

**Fig. 3:**
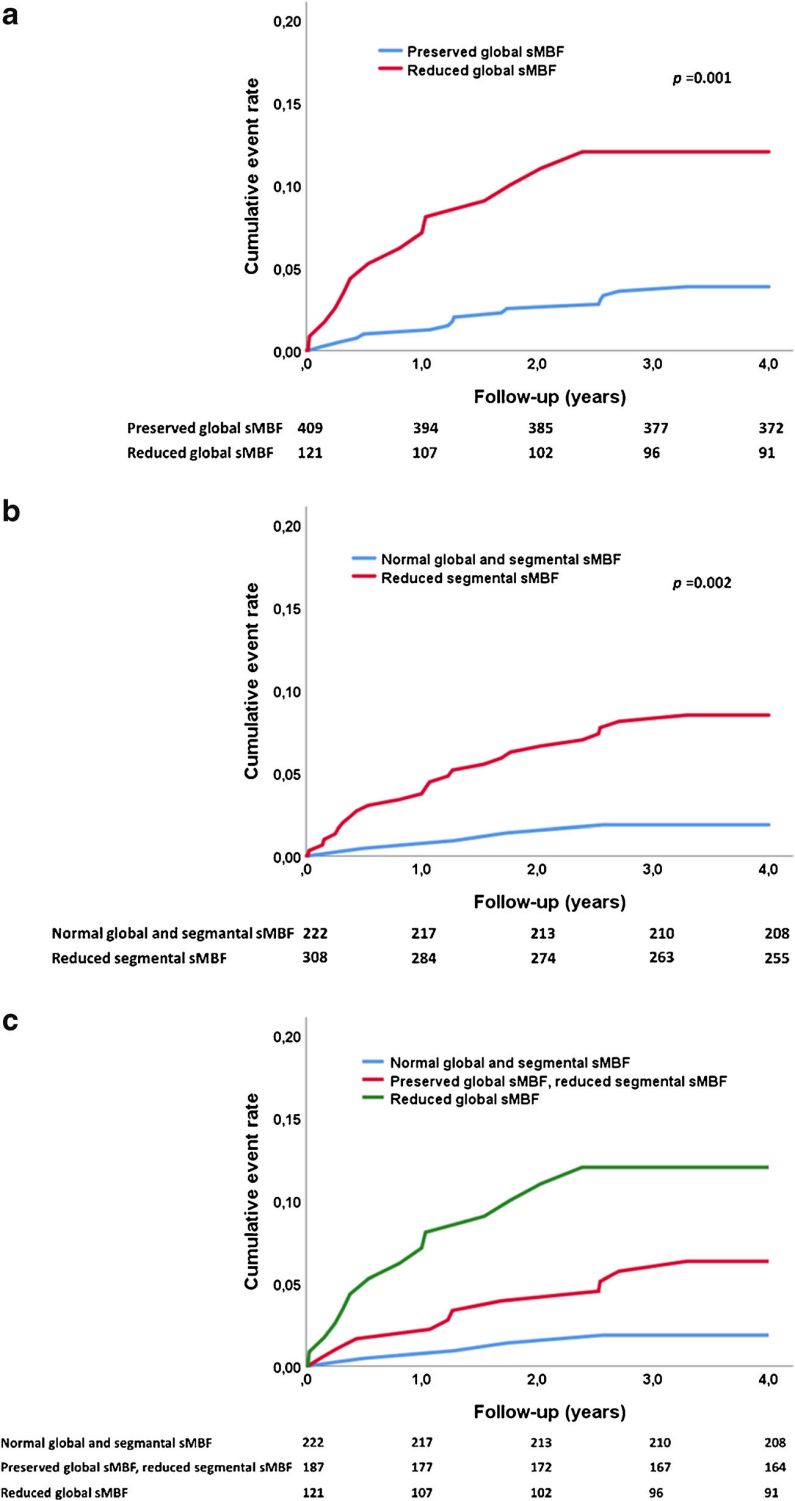
Kaplan-Meier curves showing cardiac events (cardiovascular death, myocardial infarction, or unstable angina pectoris) during 4-year follow-up according to global stress myocardial blood flow (sMBF; **a**), segmental sMBF (**b**), and combination of global and segmental sMBF (**c**)

The Kaplan-Meier estimates in Fig. 3 show that the cardiac event rate was higher in patients with reduced global sMBF than those with preserved global sMBF, with cumulative event rates at 4 years of 10.7% vs. 3.7%, respectively (log-rank *p* = 0.001, Fig. 3a). Similarly, patients with any segmental reduction of sMBF had a higher event rate than those with normal sMBF in all segments (7.8% vs. 1.8%, log-rank *p* = 0.002, Fig. 3b). There was a step-wise increase in the cumulative event rate from patients with normal sMBF in all segments, through those with reduced segmental sMBF but preserved global sMBF, and to those with reduced global sMBF (log-rank *p* = 0.001) (Fig. 3c). Patients with reduced segmental sMBF but preserved global sMBF had an event rate of 5.9% at 4 years.

### Predictors of cardiac events

Based on the Cox regression analysis, reduced global sMBF < 2.2 ml/g/min was associated with a hazard ratio of 3.191 (95% CI 1.518–6.707, *p* = 0.002) for any cardiac event at 4 years, compared with preserved global sMBF (Table 2). Any segmental reduction of sMBF was associated with a hazard ratio of 4.585 (95% CI 1.591–13.216, *p* = 0.005), compared with normal sMBF in all segments (Table 2). In univariable analysis, other predictors of cardiac events at 4 years were age (*p* = 0.003), diabetes (*p* = 0.008), and number of segments with reduced sMBF (*p* = 0.014, Table 2).

The independent predictors of cardiac events at 4 years in different multivariable regression models including age and diabetes as clinical risk factors are shown in Table 3. When adjusted for age and diabetes, the number of segments with reduced sMBF was a predictor of adverse events (HR 1.082, 95% CI 1.021–1.146, *p* = 0.008), but it lost significance when global reduction of sMBF was added to the model (*p* = 0.8). Both reduced global sMBF and any segmental reduction of sMBF remained as independent predictors of cardiac events at 4 years when adjusted for other clinical variables. Patients with reduced global sMBF had a 7-fold incremental hazard of 4-year cardiac events (HR 6.970, 95% CI 2.271–21.396, *p* = 0.001), whereas those with preserved global sMBF combined with reduced segmental sMBF had a 3-fold incremental hazard, over those with normal sMBF in all segments (HR 3.251, 95% CI 1.030–10.257, *p* = 0.044). In that latter regression model, patients with reduced global sMBF had a 2-fold incremental hazard (HR 2.146, 95% CI 0.956–4.808, *p* = 0.06) over those with reduced segmental sMBF but preserved global sMBF.

**Table 3:** Independent predictors of cardiac events at 4 years in different Cox regression models

Multivariable model	HR, 95%CI	*p* value
Clinical variables only		
Age (years)	1.072, 1.024–1.123	0.003
Diabetes	2.746, 1.286–5.863	0.009
Clinical variables and reduced global sMBF		
Age (years)	1.072, 1.025–1.122	0.002
Diabetes	2.854, 1.335–6.098	0.007
Reduced global sMBF	3.439, 1.634–7.235	0.001
Clinical variables and reduced segmental sMBF		
Age (years)	1.076, 1.028–1.127	0.002
Diabetes	2.477, 1.158–5.296	0.019
Reduced segmental sMBF	4.600, 1.592–13.298	0.005
Clinical variables, global sMBF, and segmental sMBF		
Age (years)	1.075, 1.027–1.124	0.002
Diabetes	2.648, 1.234–5.680	0.012
Preserved global sMBF, but reduced segmental sMBF	3.251, 1.030–10.257	0.044
Reduced global sMBF	6.970, 2.271–21.396	0.001

*CI* confidence interval, *HR* hazard ratio, *sMBF* stress myocardial blood flow

### Incremental prognostic value of global and segmental sMBF

Models describing the prognostic value of different PET parameters compared with clinical risk predictors are shown in Table 4. The addition of reduced global sMBF to other clinical risk factors resulted in a significant incremental predictive value for cardiac events at 4 years; the model IDI was 0.034 (95% CI 0.006–0.062, *p* = 0.016) and continuous NRI 0.498 (95% CI 0.118–0.879, *p* = 0.010) (Table 4). Likewise, the addition of any segmental reduction of sMBF to other clinical risk factors resulted in a significant incremental predictive value for a 4-year primary outcome; the model IDI was 0.028 (95% CI 0.014–0.043, *p* = 0.0001) and continuous NRI 0.583 (95% CI 0.203–0.963, *p* = 0.002).

**Table 4:** Net reclassification improvement and integrated discrimination improvement according to different myocardial sMBF patterns compared to clinical variables only

Multivariable model	NRI, 95% CI	*p* value	IDI, 95% CI	*p* value
Clinical variables and reduced global sMBF	0.498, 0.118–0.879	0.010	0.034, 0.006–0.062	0.016
Clinical variables and reduced segmental sMBF	0.583, 0.203–0.963	0.002	0.028, 0.014–0.043	0.0001

*CI* confidence interval, *IDI* integrated discrimination improvement, *NRI* net reclassification improvement, *sMBF* stress myocardial blood flow

## Discussion

The current study demonstrated that both global and segmental sMBF detected by [^15^O]water PET predicted cardiac events including cardiovascular death, MI, or unstable angina at 4-year follow-up in symptomatic patients evaluated for suspected obstructive CAD and having coronary stenosis on coronary CTA. Global sMBF < 2.2 ml/g/min was the cutoff value that best predicted events.

Patients with reduced global sMBF carried a 7-fold adjusted hazard of the composite adverse outcome at 4 years compared to those who had normal sMBF in all segments, whereas those who had preserved global sMBF combined with segmentally reduced sMBF carried a 3-fold such hazard. Both the reduced global sMBF and the presence of any segmental reduction of sMBF provided similar incremental prognostic value for the prediction of cardiac events over clinical risk predictors.

The independent prognostic value of absolute measures of MBF for the prediction of cardiac adverse events at long-term follow-up has been demonstrated in several studies [14–21, 26–28]. However, there is a significant heterogeneity in studies in terms of risk estimates, patient populations, and measures of MBF [14]. In particular, the current evidence is mainly based on global MFR by ^82^Rb or [^13^N] ammonia PET, whereas there is limited data on the prognostic value of [^15^O]water PET, the use of sMBF instead of MFR, and the relative impact of regional and global sMBF on the outcomes.

There is some inconsistency in the evidence supporting the prognostic role of reduced sMBF that does not differentiate between irreversible and reversible perfusion abnormalities. We have previously demonstrated that abnormal regional sMBF by [^15^O]water PET (defined as sMBF ≤ 2.3 ml/g/min) independently predicted composite adverse outcomes including all-cause mortality, MI, and unstable angina, over a median follow-up of 3.6 years in patients without previous MI [21]. The predictive value of global sMBF was not addressed in that study, but Farhad et al. observed that reduced global sMBF and MFR by ^82^Rb PET independently predicted a broader composite outcome including also revascularization and hospitalization [17]. By contrast, in another study with a relatively short follow-up, age-adjusted global MFR (< 2.1) independently predicted a composite outcome including both “hard” and “soft” adverse events, while global sMBF (< 1.9 ml/g/min) did not [18]. In a large cohort, Gupta et al. reported that global MFR was a stronger predictor of cardiovascular mortality than impaired sMBF at a median follow-up of 5.6 years [16]. Adjusted cardiovascular mortality was independently driven by MFR, irrespective of whether the sMBF was impaired or preserved [16].

Studies have shown that global MFR provides prognostic information that is independent of the presence of regional myocardial ischemia [15, 16, 20]. In these studies using ^82^Rb or [^13^N] ammonia PET, the presence of regional ischemia was based on visual or semiquantitative evaluation of relative perfusion defects (either the summed stress score or the combined extent of myocardial scar and ischemia) instead of regional absolute values of MBF. A recent study by Bom et al. was the first to explore the relative prognostic value of global and regional absolute MBF determined by [^15^O]water PET [29]. The investigators found that both global and regional sMBF had similar prognostic value in predicting the composite of death and MI, while the combination of global and regional sMBF did not improve the prognostic performance compared to either alone. Moreover, in adjusted analyses, sMBF but not MFR remained an independent predictor of outcome. Our findings are generally in line with the observations by Bom et al. [29], although some differences between these studies exist. Importantly, the optimal cutoff point of global sMBF identified by Bom et al. [29] was considerably higher (2.65 ml/g/min) compared to the optimal cutoff point identified in our study (2.2 ml/g/min). Interestingly, our study demonstrated 2.7 ml/g/min as a second-best prognostic cutoff for global sMBF, while we decided to adopt the cutoff 2.2 ml/g/min to optimize specificity.

The clinical utility of a risk marker is reflected by its ability to reclassify the risk of adverse events when added to a baseline clinical risk model; such a risk reclassification might potentially inform clinical decision-making to influence patient management and consequently improve outcome. In this sense, the novel metrics of risk reclassification such as the NRI and the IDI are useful in determining the clinical utility of a novel risk marker. The current study showed that the presence of either reduced global sMBF or reduced segmental sMBF in [^15^O]water PET provides a significant improvement in the risk reclassification over clinical risk factors (large effect size with a continuous NRI of 0.498 and 0.583, respectively). A relatively small number of patients had globally reduced sMBF (23% of cohort), and the absolute numbers of events were similar in patients with globally reduced sMBF and patients with segmental reduction of sMBF but normal global sMBF (13 vs. 11, respectively) in this relatively low-risk cohort with an annual event rate of 1.3%.

Differences in the prognostic value of various sMBF parameters may be related to different disease phenotypes. Patients with impaired global sMBF despite low regional ischemic burden can be considered to represent predominantly non-obstructive CAD or microvascular disease [16]. The patients with regionally reduced sMBF but preserved global sMBF represent patients with limited regional ischemia, and the patients with both global and regional reduced sMBF are likely those with extensive ischemic CAD or severe microvascular disease. When using only globally reduced sMBF as a predictor of outcome, patients with regionally limited ischemia that is not large enough to cause global reduction are missed. On the other hand, using only regional ischemia as a predictor of outcome, the extent of myocardial ischemia is ignored.

### Limitations

The current study has all the inherent limitations of the retrospective observational study design. The current study enrolled a comparatively low-risk cohort with preserved left ventricle function, yet the consecutive enrollment of the patients in our registry is a strength of this study because the cohort is representative of real-life patients with suspected CAD referred for a diagnostic work-up.

It is worth noting that in our center, PET perfusion imaging is performed selectively after coronary CTA, and consequently, the current prognostic analysis included a selected cohort of patients with CTA suggestive of obstructive CAD. Thus, patients with microvascular dysfunction in the absence of coronary atherosclerosis or in the presence of non-obstructive atherosclerosis were not included, because perfusion imaging is not performed in such cases. However, we have previously demonstrated in a similar patient population that the prevalence of microvascular dysfunction is relatively low in the absence of coronary atherosclerosis or in the presence of non-obstructive coronary atherosclerosis (1% and 3%, respectively) in such patient population [30]. Considering patient characteristics and previous studies showing that reduced sMBF by [^15^O]water PET accurately detects obstructive CAD [5, 7, 10, 22], our results suggest that globally reduced sMBF identifies patients with extensive CAD and thus may provide risk-based guidance for triage to invasive coronary angiography. The identified optimal cutoff value (< 2.2 ml/g/min) of global sMBF was based on our selected cohort and might not be applicable to other centers in which different methodology is employed. The limited size of our cohort did not allow exploring the impact of revascularization on survival, but recent studies have provided evidence that reduced global MFR or severely reduced regional sMBF by ^82^Rb PET are associated with a survival benefit gained by early revascularization [27, 28].

The small number of events precluded comparison of sMBF with many covariates, such as the extent of coronary atherosclerosis and atherosclerotic plaque characteristics that may provide additional risk stratification. Finally, our data are derived from a single-center which implements a standard sequential hybrid imaging protocol and employs stress-only [^15^O]water PET that precludes direct comparison of MFR and sMBF. Despite its limitations, the current study builds up more evidence in favor of the prognostic value of reduced sMBF at both global and segmental levels for the prediction of cardiac events at a relatively long follow-up period.

## Conclusion

In symptomatic patients evaluated for suspected obstructive CAD, reduced global absolute sMBF by [^15^O]water PET identifies those at the highest risk of cardiac events, whereas segmental reduction of sMBF with preserved global sMBF is associated with an intermediate event risk. Reduced global sMBF and segmental sMBF were associated with similar risk reclassification over clinical risk factors.

## Data Availability

Please contact the authors for data requests.
